# Neuroprotective Effect of siRNA Entrapped in Hyaluronic Acid-Coated Lipoplexes by Intravitreal Administration

**DOI:** 10.3390/pharmaceutics13060845

**Published:** 2021-06-08

**Authors:** Marcela Coelho Silva Ribeiro, Marcelo Coutinho de Miranda, Pricila da Silva Cunha, Gracielle Ferreira Andrade, Gustavo de Oliveira Fulgêncio, Dawidson Assis Gomes, Sílvia Ligorio Fialho, Frederico Pittella, Christine Charrueau, Virginie Escriou, Armando Silva-Cunha

**Affiliations:** 1 Faculdade de Farmácia, Universidade Federal de Minas Gerais, 31270-901 Belo Horizonte, Brazil; marcelacsribeiro@gmail.com (M.C.S.R.); fulgenciobr@yahoo.com.br (G.d.O.F.); 2 Université de Paris, CNRS, INSERM, UTCBS, Unité des Technologies Chimiques et Biologiques pour la Santé, 75006 Paris, France; christine.charrueau@parisdescartes.fr (C.C.); virginie.escriou@parisdescartes.fr (V.E.); 3 Departamento de Bioquímica e Imunologia, Instituto de Ciências Biológicas, Universidade Federal de Minas Gerais, 31270-901 Belo Horizonte, Brazil; marcelocdem@gmail.com (M.C.d.M.); pribqi@yahoo.com.br (P.d.S.C.); dawidson.gomes@gmail.com (D.A.G.); 4 Departamento de Ciências da Saúde, Universidade Federal do Espírito Santo, 29932-540 São Mateus, Brazil; graciellefandrade@yahoo.com.br; 5 Diretoria de Pesquisa e Desenvolvimento, Fundação Ezequiel Dias, 30510-010 Belo Horizonte, Brazil; silvia.fialho@funed.mg.gov.br; 6 Departamento de Ciências Farmacêuticas, Universidade Federal de Juiz de Fora, 36036-900 Juiz de Fora, Brazil; frederico.pittella@ufjf.edu.br

**Keywords:** lipoplexes, hyaluronic acid, siRNA, caspase-3, retinal neuroprotection, intravitreal administration

## Abstract

Since the possibility of silencing specific genes linked to retinal degeneration has become a reality with the use of small interfering RNAs (siRNAs), this technology has been widely studied to promote the treatment of several ocular diseases. Despite recent advances, the clinical success of gene silencing in the retina is significantly reduced by inherent anatomical and physiological ocular barriers, and new strategies are required to achieve intraocular therapeutic effectiveness. In this study, we developed lipoplexes, prepared with sodium alginate as an adjuvant and strategically coated with hyaluronic acid (HA-LIP), and investigated the potential neuroprotective effect of these systems in a retinal light damage model. Successful functionalization of the lipoplexes with hyaluronic acid was indicated in the dynamic light scattering and transmission electron microscopy results. Moreover, these HA-LIP nanoparticles were able to protect and deliver siRNA molecules targeting caspase-3 into the retina. After retinal degeneration induced by high light exposure, in vitro and in vivo quantitative reverse transcription-PCR (RT-qPCR) assays demonstrated significant inhibition of caspase-3 expression by HA-LIP. Furthermore, these systems were shown to be safe, as no evidence of retinal toxicity was observed by electroretinography, clinical evaluation or histology.

## 1. Introduction

Around 285 million people are visually impaired in the world, suffering from ocular diseases such as age-related macular degeneration (AMD), diabetic retinopathy, uveitis and glaucoma. Moreover, the number of patients suffering from chronic and progressive blinding retinal disorders is increasing due to the aging population. Currently, significant functional recovery for most of the retinal degeneration diseases has not been achieved in clinical practice and remains a major challenge. Therefore, new drug delivery strategies are required to achieve therapeutic effectiveness by overcoming the physiological barriers in the eyes [1,2].

RNA interference (RNAi) has recently advanced as a powerful tool to promote specific gene regulation and it is a promising technique to improve therapeutic outcomes in ophthalmic applications [3,4,5]. Among the silencing molecules, small interfering RNAs (siRNAs) stand out, as they do not follow Dicer processing, avoiding the overload of the cellular RNAi path. The siRNA molecule is a double-stranded RNA oligonucleotide, usually with 21–27 base pairs [6,7,8]. The eye appears particularly attractive for RNAi therapies because it is easily accessible, relatively immune-privileged (due to the limited systemic exposure promoted by the blood–retina barrier (BRB)) and small, which allows small amounts of agent to achieve a therapeutic effect [9,10,11]. In addition, the monogenic nature of retinal diseases and the current knowledge of their molecular pathogenesis favors research on gene-related therapeutic approaches. Until now, more than 260 genes and loci related to retinal diseases such as RPE65-Leber congenital amaurosis (RPE65-LCA) and X-linked juvenile retinoschisis have been mapped in a genetic database [5,12].

The intraocular delivery of siRNA is limited by several inherent anatomical and physiological ocular structures, including the cornea and anterior segment barriers, the sclera and Bruch’s choroid complex, as well as the BRB [13,14]. In addition, siRNA molecules cannot freely cross cell membranes because of their relatively large molecular weight and phosphodiester backbone [6,7,8]. Thus, the effectiveness of RNAi therapy significantly depends on the siRNA vector’s ability to overcome the intrinsic ocular barriers and reach the target cells [3,15,16].

Viral vectors and non-viral vectors are two main approaches used for siRNA or gene transfer in ocular applications [17,18]. The most used viral vectors for retinal gene transfer are those derived from adenoviruses, lentiviruses and adeno-associated viruses, which can infect and transduce non-dividing cells, such as photoreceptors and the retinal pigment epithelium (RPE) [12,19,20,21]. However, viral vectors carry the inherent risk of oncogenicity and immunogenicity. On the other hand, there is an increasing interest in developing non-viral delivery systems such as lipoplexes to protect siRNA from degradation and facilitate its endocytosis by retinal cells [5,7,22,23]. Generally, lipoplexes are formed by the spontaneous binding of nucleic acids to cationic liposomes. However, recent studies using ex vivo assays demonstrated that the positive charge of the cationic lipoplex could be detrimental for its mobility and induce aggregation into the vitreous [24,25]. Moreover, a thin layer of the vitreous on top of retinal cells almost completely blocks classical cationic lipoplexes from delivering nucleic acids [26,27].

One promising strategy to reach the retina is coating cationic liposomes with an anionic polymer such as hyaluronic acid (HA). HA is composed of disaccharide units of N-acetylglucosamine and glucuronic acid chains alternately, β1→4 and β1→3 [28,29]. This biopolymer has good biocompatibility and biodegradability, and it is naturally present in the eye as a major constituent of the vitreous humor. Moreover, it plays an essential role in cell signaling and can bind cell membranes via CD44 receptors, expressed on the surface of hematopoietic, neuronal and epithelial cells [29,30]. The aim of this work was to investigate the safety and efficiency of an HA-coated cationic lipoplex (HA-LIP) to deliver caspase-3 siRNA in retinal neurodegeneration rat models by retinal light injury.

## 2. Materials and Methods

### 2.1. Cell Culture and Animals

The ARPE-19, a human retinal pigment epithelial cell line, was cultured in Dulbecco’s Modified Eagle Medium, Nutrient Mixture F-12 (DMEM/F-12, Life Technologies, Carlsbad, CA, USA), supplemented with 2.43 g/L sodium bicarbonate (Sigma-Aldrich, St. Louis, MO, USA), 1% penicillin/streptomycin/amphotericin B solution (PSA; Sigma-Aldrich) and 10% fetal bovine serum (Gibco). Cells were cultivated in a humid atmosphere at 37 °C and 5% CO_2._ The culture media was changed every 2 days.

Adult male Wistar rats (180–200 g) were used. The rats were housed in a 12-h light-dark cycle. Animals received food and water ad libitum. All experiments were conducted in accordance with the guidelines of the Association for Research in Vision and Ophthalmology (ARVO). The ethics protocol was approved by the Ethics Committee on the Use of Animals of Federal University of Minas Gerais (Comissão de Ética no Uso de Animais, CEUA-UFMG; date of approval: 8 April 2019; approval number: 31/2019).

### 2.2. siRNAs

The non-targeting siRNA used as control (5′ UUC UCC GAA CGU GUC ACG UdTdT 3′) and Rhodamine-labelled siRNA were purchased from Eurogentec (Seraing, Belgium). For the gene silencing experiments, different Stealth siRNA sequences targeting caspase-3 were obtained from Invitrogen (Life Technologies, Carlsbad, CA, USA).

Stealth siRNA 371 or 373 is a 25-bp duplex oligoribonucleotide with a sense strand corresponding to human *CASP3* mRNA sequence, while Stealth siRNA 565 or 566 corresponds to rat *Casp3* mRNA sequence. The Stealth siRNA sequences are shown in Table 1.

### 2.3. Liposomes and Lipoplexes

Cationic liposomes were composed of DMAPAP cationic lipid (RPR 209120 2-{3-[bis-(3-amino-propyl)-amino]-propylamino}-N-ditetradecyl carbamoyl methyl-acetamide), synthesized as in [31], and DOPE (1,2-dioleoyl-sn-glycero-3-phosphoethanolamine), obtained from Avanti Polar Lipids (Alabaster, AL, USA). Cationic liposomes were prepared as reported previously by our research group [6,32].

Lipoplexes were prepared by mixing siRNA (1.5 µg) and a sodium alginate (Sigma-Aldrich, Saint Quentin Fallavier, France) solution at ratio of 1:1 *(w/w*) in 150 µL of NaCl 150 mM, with an equal volume of the cationic liposome suspension also prepared in 150 mM NaCl. Lipoplexes were allowed to form for 30 min at room temperature before use. The charge ratio of cationic lipoplexes was R+/− = 8, calculated using the molar ratio of positive charges (positive charges per molecule of DMAPAP) to the molar ratio of negative charges (from siRNA and anionic polymer molecules: respectively, 3.03 nmol and 5.05 nmol of negative charges/mg for siRNA and sodium alginate). For coating lipoplexes with hyaluronic acid (HA-LIP), the cationic lipoplexes were rapidly mixed by vortexing with an equal volume of a hyaluronic acid (160–600 kDa, bio-sodium hyaluronate powder) solution, 0.3% (*w/v*).

### 2.4. Physicochemical and Morphological Characterization of Lipoplexes

The hydrodynamic diameter (intensity-weighted Z-average) and polydispersity index (PdI) of the cationic lipoplex and HA-LIP were measured by dynamic light scattering (DLS) with a Zetasizer NanoZS (Malvern Instruments, Malvern, UK) with a He-Ne laser (633) as the incident beam and a detection angle of 173°. Zeta potential (ZP) measurements were performed using the electrophoretic mobility technique in a Zetasizer NanoZS (Malvern Instruments, UK). The amount of siRNA encapsulated inside the liposome preparations was quantified by a RiboGreen assay using a siRNA standard curve, according to the manufacturer’s instructions. The encapsulation efficiency was calculated by determining unencapsulated siRNA content and comparing its fluorescence value with the total siRNA content that was obtained upon lysis of the lipoplex treatment with an NaCl 1 M solution and 10% Triton X 100 (Sigma-Aldrich). Measurements were done at wavelengths of 480/525 nm.

For TEM, 3 µL of each formulation was placed on 400 mesh coated copper grids (Lacey Formvar/Carbon; Electron Microscopy Sciences, Hatfield, PA, USA) and allowed to adsorb, and the excess amount of liquid was removed with a filter paper. Negative staining was made with uranyl acetate and preparations were analyzed at room temperature on a Tecnai Spirit 120 electron microscope (Thermo Fisher Scientific, Hillsboro, OR, USA) operating at accelerating voltage of 120 kV under a low electron dose.

For cryo-TEM analysis, cryo-TEM grids were prepared using a GPM2 Plunge Freezer (Leica, Wetzlar, Germany). Specimens were prepared in a controlled environment with the temperature and humidity set to 22 °C and 100%, respectively, which prevented sample evaporation during preparation. A 3 μL sample droplet was deposited on a 400 mesh lacey carbon-coated copper grid (EMS) and prepared with a blot time of 3 s. Specimens were analyzed in low dose conditions on a Tecnai Spirit 120 electron microscope (Thermo Fisher Scientific, Hillsboro, OR, USA) operating at an accelerating voltage of 120 kV.

### 2.5. Gel Retardation Assay

Uncoated and HA-coated lipoplexes were prepared in 150 mM NaCl containing 0.3 μg siRNA/sample (in 10 μL). The same concentration of naked siRNA was used as a control. Samples were treated with a NaCl 1 M solution and 10% Triton X 100 (Sigma-Aldrich) for 20 min at 25 °C to induce siRNA release. Afterward, 10 μL of a TBE–urea sample buffer 2× was added to the samples and they were electrophoresed on 8% polyacrylamide gels in a TBE buffer, running at 90 V for 40 min. After migration, the gel was stained with ethidium bromide for 30 min. The stained siRNA bands were visualized under a UV transilluminator.

### 2.6. Lipoplex Uptake by ARPE-19 Cells

ARPE-19 cells were plated onto 6-well plates containing sterile glass slides at 1 × 10^5^ cells/well, in 2 mL of the culture medium added to the lipoplex formulation. Uncoated and HA-coated lipoplexes were prepared as described above using control siRNA (without fluorescence) or rhodamine-labelled siRNA. Lipofectamine 2000 (Thermo Fisher Scientific, Carlsbad, CA, USA) containing the same concentration of rhodamine-labelled siRNA was used as a control. Twenty-four hours after treatment, cells were washed with PBS and fixed for 10 min with 10% (*w/v*) paraformaldehyde. These cells were then incubated in the presence of PBS containing 0.1% Triton X-100 (Sigma-Aldrich) for 5 min and blocked in PBS 1% (*w/v*) bovine serum albumin (BSA, Sigma-Aldrich) and PBS 5% goat serum. In addition, the cells were double-labeled with monoclonal antibodies against α-tubulin (Sigma-Aldrich) and lamin B1 (Abcam, Cambridge, UK). Next, cells were washed with PBS and incubated with secondary antibodies conjugated to Alexa 488 and 647, followed by nuclear staining with Hoechst 33,258 (Life Technologies). Cells were washed in PBS and mounted in Prolong Gold Antifade reagent (Life Technologies). The negative control was included by omitting the primary antibodies. A Zeiss LSM 880 (Carl Zeiss, Oberkochen, Germany) confocal microscope was used to obtain the images with an oil 63 × 1.4 NA objective lens with excitation at 405, 488, 543 and 633 nm. The ZEN Black edition software (Carl Zeiss) was used to process the images.

### 2.7. Cellular Viability

The viability of ARPE-19 cells after treatment with the lipoplexes was evaluated by MTT. Briefly, the cell suspension was seeded on 24-well plates at a density of 1 × 10^5^ cells/well with uncoated or HA-coated lipoplexes, added to the cells in triplicate, containing different concentrations of siRNA: 20 nM (log 10^1.3^), 40 nM (log 10^1.60^), 60 nM (log 10^1.77^), 80 nM (log 10^1.90^) and 100 nM (log 10^2.0^). Next, the cells were incubated at 37 °C in the presence of 5% CO_2_ for 24 h. After that, the medium containing the lipoplexes was replaced by a fresh culture medium and the cells were incubated for an additional period until 48 h. The viability was evaluated by adding MTT (500µg/mL in DMEM) directly into the cell plates and incubation for 5 h. In sequence, formazan crystals were suspended in a solution of isopropanol containing 0.06 M HCl and 0.5% SDS. The color intensity of the samples was measured in a microplate reader at 570 nm and the results were expressed relative to untreated cells.

### 2.8. Intravitreal Injections and In Vivo Retinal Distribution of Lipoplex

The rats were anesthetized with an intraperitoneal injection of ketamine (80 mg/kg) and xylazine (8 mg/kg) before the intravitreal injection. A drop of 0.4% oxybuprocaine hydrochloride was also applied in each rat’s eye to perform local anesthesia of the cornea. Five microliters of lipoplexes (uncoated and HA-coated) or a physiologic saline solution was injected into the posterior side of the globe using syringes mounted with 6 mm × 31 G needles (BD).

For the retinal distribution study, the animals were divided into 2 groups, according to the lipoplex formulation. In the first group (*n* = 4), 5 μL of cationic lipoplex containing rhodamine-labelled siRNA was administered through intravitreal injections in the left eye. Similarly, the second group of animals (*n* = 4) received 5 μL of HA-LIP containing rhodamine-labelled siRNA by intravitreal injection. The contralateral eye in both groups served as a control and was injected with 5 μL of lipoplex containing control siRNA without fluorescence. Three hours after the intravitreal injections, the rats in this study were euthanized. Their eyes were enucleated and fixed immediately with a 4% paraformaldehyde solution (PFA). On the next day, these samples were washed with PBS and incubated with a sucrose solution in PBS at room temperature before inclusion in the Optimal cutting temperature compound (OCT) compound. Next, the eyes were frozen and immediately stored at −70 °C until sectioning. The 10 µm cryo-sections were prepared by using a cryostat (LeicaCM3050S, Nussloch, Germany). The Zeiss LSM 5 Live (Carl Zeiss, Oberkochen, Germany) confocal microscope was used to obtain the images of retinal tissues.

### 2.9. Electroretinogram (ERG)

ERG was performed using an Espion (Diagnosys LLC, Cambridge, UK). Before the ERG exams, rats were kept in total darkness for 12 h. After this period, animals were anesthetized by intraperitoneal injections of ketamine (80 mg/kg) and xylazine (8 mg/kg) before the exam to reduce electrical or movement interference. Pupils were dilated with 0.5% tropicamide (Midriacyl, São Paulo, Brazil). In addition, a drop of 0.4% oxybuprocaine hydrochloride was also applied to perform local anesthesia of the cornea. The contact lens electrodes (Rodent Contact Lens, Ocuscience, Henderson, NV, USA) were carefully adapted over the cornea of the animal, and the reference and ground electrodes (Stainless Steel Subdermal Needle Electrode, Ocuscience) were placed between the eyes and on the tail, respectively. During the experiment, animal preparation and ERG conditions were performed under low red light intensity.

The ERG protocol consisted of 3 steps: rod peak response, scotopic maximum response and a photopic response. Rod peak response and scotopic maximal response ERGs were elicited using stimulus intensities between 0.00003 cd.s/m^2^ and 3 cd.s/m^2^, respectively, on a dark background. After light adaptation for 10 min, photopic ERGs were elicited using 3 cd.s/m^2^ on a white background of 30 cd/m^2^, from 15 Hz until 45 Hz. For each recording, 15 separate responses were averaged in the ERG recording system, including the amplitude and latency of each waveform. All procedures were performed in accordance with the standards recommended by the International Society of Clinical Electrophysiology of Sight (ISCEV).

Electrophysiological measurements were performed after the intravitreal injections, at 7 and 14 days in 2 groups of rats: 1 group that received 5 μL of cationic lipoplex (*n* = 4) in the left eyes and the other group, which received HA-LIP. The contralateral eyes in both groups served as controls and were injected with 5 μL of sterile physiological saline.

### 2.10. Clinical and Histological Evaluation

The indirect ophthalmoscopy (Omega 500 Binocular Indirect Ophthalmoscopy, Heine Optotechnik, Gilching, Germany) was done before the intravitreal injections and afterwards at the predetermined intervals of 7 and 14 days. In addition, the fundus examination was performed using a 90 D lens (Wlch Allyn, Skaneateles Falls, NY, USA). The intraocular pressure (IOP) of both eyes of each rat was assessed by a portable tonometer Tonopen XL (Reichert New York, NY, USA). Before IOP measurements, each eye was anesthetized in the cornea with a drop of 0.5% proxymetacaine hydrochloride (Anestalcon; Alcon, São Paulo, Brazil). To reduce variations, IOP was assessed between 12:00 p.m. and 13:00 p.m. each day or week. Three IOP readings were obtained from each eye and these results were expressed as the group mean IOP ± SD.

After the last ERG recording on Day 14, the rats were euthanized (*n* = 8) and their eyes were enucleated and immediately fixed in Karnovsky’s fixative (cacodylate buffer: 0.1 M, pH 7.4). These eyes were then rinsed in a cacodylate buffer (cacodylate buffer: 0.1 M, pH 7.4) and dehydrated in an ascending series of alcohols to be embedded in paraffin. Tissues sections of 4 μm were cut and stained with hematoxylin and eosin for light microscopy investigation (Zeiss, Model Axio Imager M2).

### 2.11. Light-Induced Retinal Degeneration

One day before the in vitro retinal light damage, HA-LIP containing different human Stealth siRNA sequences targeting *CASP3* (siRNA 371 or 373) were incubated at 37 °C with the ARPE-19 cells at 1 × 10^5^ cells/well, in triplicate. After 24 h, these cells were exposed to white LED lights (Cool LED flashlights, 3800 lumens, 7 W, Sanyi, Guangzhou, China), with wavelengths of 460–475 nm, at 2500 lux. The light source was situated 5 cm below the cell’s plaques for 8 h. Next, the medium containing the lipoplexes was replaced by a fresh culture medium, and the cells were incubated for an additional period of 24 h. After 48 h of transfection, gene expression was evaluated by quantitative reverse transcription-PCR (RT-qPCR).

In vivo retinal light degeneration was performed in a light box equipped with white LED lamps (Cool LED Lamps 6000 K, 9 W, Syflar, São Paulo, Brazil), with wavelengths of 460–475 nm, and mirrors. Briefly, the rats were placed individually in standard transparent cages, with the lamps situated 10 cm in front of the rats. Illumination was measured at the level of the eyes of the rats by a lux meter (model MLM-1011, Minipa, Houston, TX, USA). The animals received programmed LED exposure at 40,000 lux for 8 h. One day before the retinal light degeneration, the rats were anesthetized with an intraperitoneal injection of ketamine (80 mg/kg) and xylazine (8 mg/kg), followed by the intravitreal injection of 5 μL of HA-LIP containing Stealth siRNA sequences (565 or 566). A drop of 0.4% oxybuprocaine hydrochloride was also applied in each rat eye to perform local anesthesia of the cornea. Control experiments were performed by exposing animals only to a 12:12 h light–dark (LD) cycle, with conventional fluorescent lights, without LED light exposure. A drop of 0.5% tropicamide was applied 1 h before the LED light exposure for pupil dilation. After 48 h of the light exposure, the rats were euthanized, and their eyes were enucleated to perform 2 different assays: the Casp3 RNA expression was evaluated by RT-qPCR, and the structural modifications of the retina were examined by TEM. Thus, after rat eye enucleation, they were immediately fixed in a 4% glutaraldehyde cacodylate buffer (0.1 M, pH 7.4), then these samples were additionally fixed in 1% osmium tetroxide in a cacodylate buffer (0.2 M, pH 7.4) and dehydrated in an ascending series of alcohols before the propylene oxide addition. Each area of interest was included in epoxy resin, and ultrathin sections were obtained with an ultramicrotome (Leica, Wetzlar, Germany) and contrasted by uranyl acetate to be analyzed with a Tecnai Spirit 120 electron microscope (Thermo Fisher Scientific, Hillsboro, OR, USA).

### 2.12. Quantitative Reverse Transcription-PCR (RT-qPCR)

After the retinal light injury, the control, untreated and treated samples of in vitro (*n* = 3) and in vivo (*n* = 3) experiments were subjected to total RNA extraction using TRIzol reagent (Invitrogen, USA), followed by treatment with RQ1 RNase-Free DNase (Promega, Madison, WI, USA) and cDNA synthesis using High-Capacity cDNA Reverse Transcription kit (Applied Biosystems, Bedford, MA, USA), as described by the manufacturers. For each condition of the in vivo assay, retinal samples were pooled from 4 rat eyes. The primers used in the RT-qPCR assays are described in Table 2.

GoTaq qPCR Master Mix 2X (Promeg, Madison, WI, USA) was used to perform the qPCR assay, according to the manufacturer’s instructions. Standard curves were generated with a series of log dilutions of cDNA to calculate the amplification efficiency (*ACTB_Human*: Eff = 93%, R^2^ = 0.99; *CASP3_Human*: Eff = 106.7%, R^2^ = 0.99; *Actb_Rattus*: Eff = 88.2%, R^2^ = 0.99; *Casp3_Rattus*: Eff = 87.1%, R^2^ = 0.99). The qPCR reactions were performed using the ABI PRISM 7900HT Sequence Detection System (Applied Biosystems, Waltham Massachusetts, USA), and the data were processed by SDS Software, version 2.4 (Applied Biosystems, Waltham Massachusetts, USA). Data represent 3 independent biological experiments with 3 technical replicates. The calculation of gene expression was performed via the Pfaffl method, and the control group was used as a calibrator in data analysis [33].

### 2.13. Data Analysis

The statistical analyses were performed using GraphPad Prism software (GraphPad Software Inc. 5.01, La Jolla, CA, USA). All the data were expressed as the mean ± standard deviation (SD) and statistical differences were analyzed using 2-way analysis of variance (ANOVA) and Tukey’s post-test. Results with *p* < 0.05 were considered significant.

## 3. Results

### 3.1. Lipoplex Characterization

To modify the surface of liposomal siRNA delivery systems, cationic lipoplexes were rapidly mixed by vortexing with the HA solution. The obtained nanoparticles were characterized in terms of mean diameter, polydispersity index (PdI), zeta potential and encapsulation efficiency (EE) (Table 3). Cationic lipoplexes presented a size and zeta potential of 133.5 ± 1.2 and + 71.1 ± 3.0 mV, respectively. In contrast, HA-LIP mean size increased by about 66% compared with the uncoated lipoplex, and surface potential decreased from 71.1 ± 3.0 mV to -34.2 ± 1.4 mV. These results were reproducible and indicated an electrostatic binding between HA and the lipoplex. Moreover, according to the RiboGreen assay results, the siRNA complexation with liposomes in both formulations achieved an encapsulation level of more than 90%.

**Figure 1:**
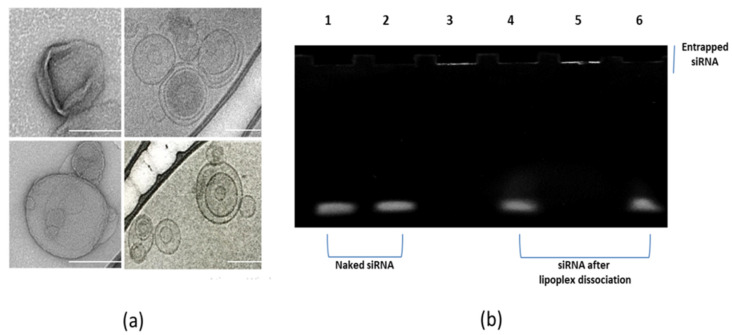
(**a**) TEM and cryo-TEM images of cationic lipoplex and HA-LIP (scale bar: 100 nm). (**b**) Acrylamide gel electrophoresis analysis of naked siRNA and lipoplexes, covered or not covered with hyaluronic acid. Each sample contained 0.3 μg of siRNA, electrophoresed through 8% acrylamide gel at 90 V for 40 min. (1) Band of intact naked siRNA; (2) naked siRNA after treatment with Triton X-100 and the NaCl 1 M solution; (3) siRNA complexed in the cationic lipoplex without treatment; (4) band of siRNA released from cationic lipoplex after treatment for dissociation of these particles; (5) siRNA complexed in the HA-LIP without treatment; (6) band of siRNA released from HA-LIP after treatment for dissociation of these particles.

TEM and cryo-TEM images confirmed the results obtained by DLS, showing that cationic lipoplexes were smaller than HA-LIP and did not reach a nanoparticle size greater than 150 nm. In addition, this study revealed the spherical shape of both lipoplexes, but HA-LIP exhibited more multivesicular liposomes than cationic systems (Figure 1a). In addition, an assessment of the siRNA complexation with cationic or HA-LIP was performed by electrophoresis on acrylamide gel (Figure 1b). For this, naked siRNA bands were compared with the siRNA bands released from these nanoparticles after the treatment with Triton X-100 and NaCl 1 M. One sample of naked siRNA also received this treatment to compare it with lipoplexes under the same conditions (Figure 1b: band 02). According to the results, both lipoplexes were able to complex with siRNA and maintain nucleic acid binding in these experimental conditions when Triton and NaCl were not added (Figure 1b: band 03 and band 05). However, after treatment, in both systems, dissociated and released siRNA could be observed as a band on the acrylamide gel–cationic lipoplex (Figure 1b: band 04) and HA-LIP (Figure 1b: band 06).

### 3.2. In Vitro siRNA Delivery and Cell Viability

**Figure 2:**
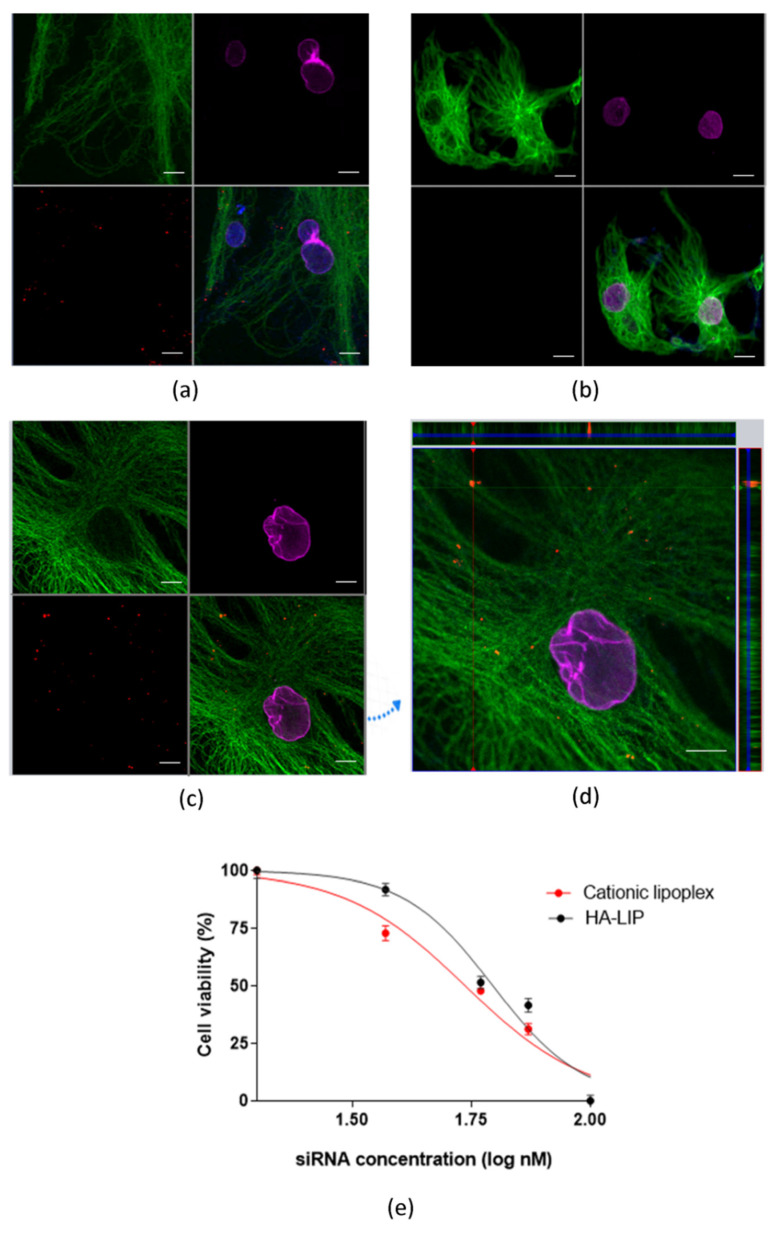
(**a**) Cellular uptake of rhodamine-siRNA complexed to the cationic lipoplex. (**b**) Cellular uptake of the negative control (absence of fluorescence) or (**c**) rhodamine-siRNA complexed to HA-LIP. (**d**) Rhodamine-siRNA internalized into ARPE-19 cells, shown by orthogonal confocal images (x-z sections at the top and y-z sections at the right of the image; scale bar: 10 µm). Representative images of serial optical sections collected for three-dimensional reconstruction of: α-tubulin in green, lamin B1 in purple, nuclear Hoechst stain in blue, rhodamine-labeled siRNA in red. (**e**) Cell viability of cationic lipoplex or HA-LIP, normalized against untreated ARPE-19 cells (*n* = 3).

The uptake of lipoplexes prepared with rhodamine-labeled siRNA by ARPE-19 cells was assessed by confocal images. As shown in Figure 2, cytoplasmic red punctuated fluorescence was observed in cells treated with HA-LIP incorporating rhodamine-labeled siRNA. Cells treated with Lipofectamine containing rhodamine-labeled siRNA also presented red fluorescent signals in the cytosol (Figure 2a). The confocal orthogonal images depict the red fluorescence in two plans inside ARPE-19 cells (Figure 2b). On the other hand, no fluorescence signals were observed in cells treated with non-labeled siRNA contained in HA-LIP (Figure 2a). The cell viability of cells treated with cationic lipoplexes, HA-LIP and lipofectamine is also shown in Figure 2c. ARPE-19 cell viability significantly decreased in a dose-dependent manner for the treatment with all the non-viral vectors. HA-LIP was the least toxic among those evaluated, showing an IC50 value of 60.73 nM. In addition, the IC50 value was 54.62 nM for cationic lipoplexes and 52.50 nM for lipofectamine.

### 3.3. Retinal Distribution of Lipoplexes

**Figure 3:**
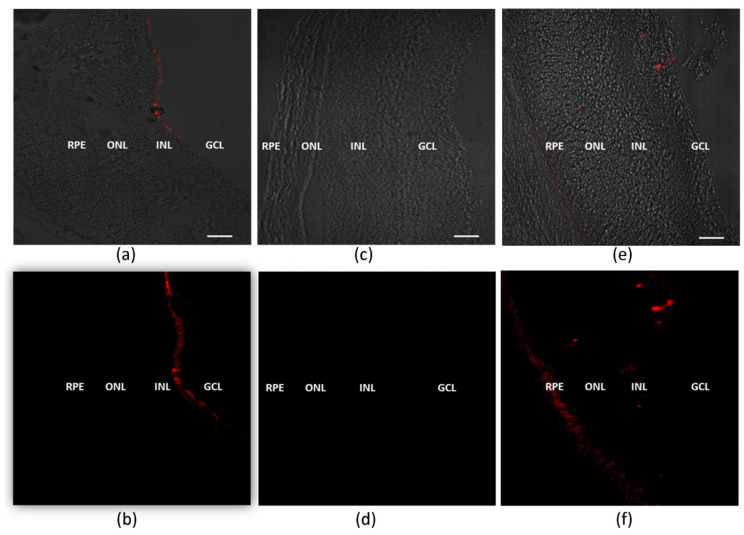
Representative fluorescence images of siRNA retinal distribution obtained from samples 3 h after intravitreal injection. The rats received intravitreal injections of cationic lipoplex containing rhodamine-labeled siRNA (**a**,**b**) and HA-LIP containing non-fluorescent siRNA (**c**,**d**) or rhodamine-labeled siRNA (**e**,**f**). The images were obtained using a Zeiss LSM 5 Live (Carl Zeiss) confocal microscope (scale bar: 30 µm). GCL, ganglion cell layer; INL, inner nuclear layer; ONL, outer nuclear layer; RPE, retinal pigment epithelium.

The distribution of both lipoplexes in the retina 3 h after intravitreal injection was indicated in confocal images by the fluorescence intensity of rhodamine-siRNA in different retinal layers (Figure 3). The siRNA delivered by the cationic lipoplex was accumulated in the ganglion cell layer and was not able to reach deeper retinal structures (Figure 3a,b). In contrast, the HA-LIP could overcome the inner limiting membrane, and the fluorescent siRNA was detected in the inner and outer nuclear layers and RPE (Figure 3e,f). No fluorescence could be detected when the control siRNA was used in contralateral eyes (Figure 3c,d).

### 3.4. Electroretinogram (ERG)

**Figure 4:**
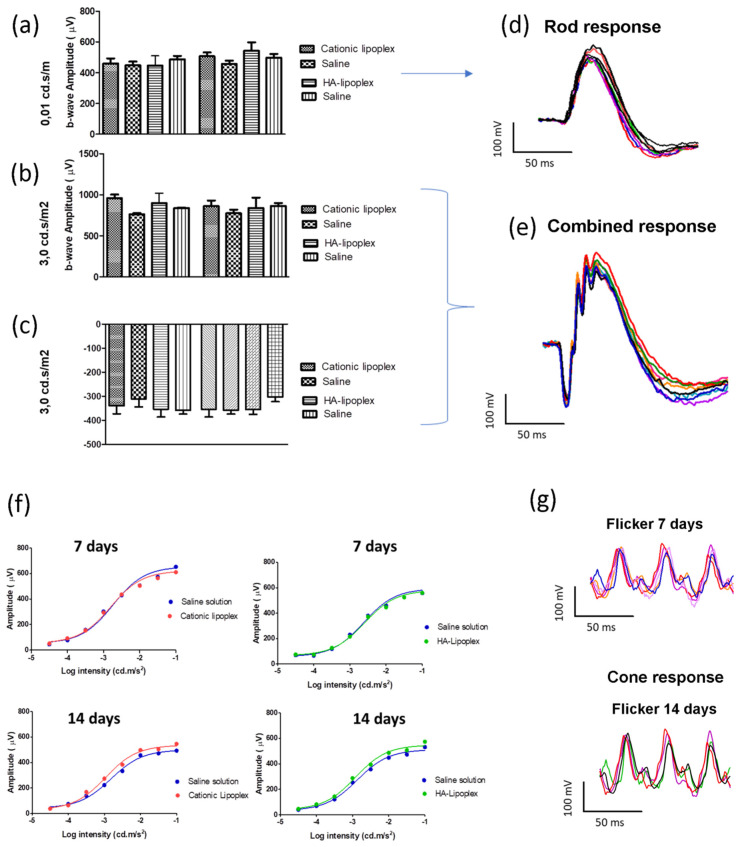
Graphical ERG measurements of dark-adapted, a- and b-waves in the experimental eyes with a stimulus of 0.01 cd.s/m^2^ and 3.0 cd.s/m^2^ (**a**–**c**). ERG was performed 7 and 14 days after the intravitreal injection. Representative ERG responses according to luminance and implicit time in dark-adapted eyes (**d**,**e**), considering the Naka–Rushton function (**f**) and light-adapted conditions (**g**).

ERG was performed 7 and 14 days after the intravitreal injections. During the scotopic condition, dark-adapted animals were exposed to increasing luminance to assess the a-wave and b-waves amplitudes and implicit times. The ERG responses are expressed in Figure 4, considering the treatment test eyes and contralateral eyes, in 0.01 cd.s/m^2^ (rod response) and 3 cd.s/m^2^ (maximal or standard combined rod–cone). In sequence, the animals were also examined in light-adapted conditions by a single-flash cone response and flicker ERG (3 cd.s/m^2^). Dark-adapted a- and b-wave amplitudes were not significantly altered in eyes that received cationic lipoplex, HA-LIP or saline during the 7 and 14 days of this experiment (*p* > 0.05, *p* = 0.5439). Moreover, similar results were found in light-adapted animals and no statistically significant differences were found between the rat eyes that received saline and those that received lipoplex (*p* > 0.05, *p* = 0.5927). The Naka–Rushton function and their relationship with increasing luminance for all groups are shown in Figure 4.

### 3.5. Clinical and Histological Evaluation

**Figure 5:**
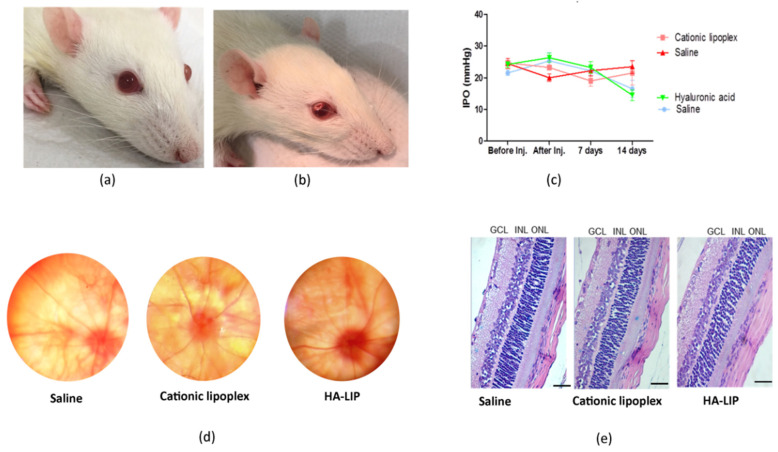
(**a**) Rat eye before the intravitreal injection and (**b**) immediately after intravitreal injection. (**c**) Rat eyes’ intraocular pressure at different times of the study (Mean ± SD). (**d**) Representative images of ocular fundus examinations after an intravitreal injection of saline, cationic lipoplex and HA-LIP. (**e**) Example of retinal sections from one rat eye subjected to intravitreal injection of physiological saline, cationic lipoplex and HA-LIP after 14 days of injections (scale bar: 50 µm). GCL, ganglion cell layer; INL, inner nuclear layer; ONL, outer nuclear layer.

No ocular damage such as corneal edema, hyperemia or conjunctival secretion, hemorrhaging, vitreous opacity or retinal detachment was observed after the intravitreal injection (Figure 5b) and during the clinical evaluation. In each animal group, ocular fundus examinations were performed and registered as representative images, shown in Figure 5d. Regarding the IOP measurements, despite the IOP variations during the experiment, these eyes had their main values in the normal range of rat IOPs (16.5 ± 5.4 for saline and 14.5 ± 3.4 for HA-LIP) (Figure 5c). Histological examination of rat retinas with light microscopy did not show any abnormality or changes in the retinal layers’ morphology (Figure 5e).

### 3.6. Reduction of Pro-Apoptotic Caspase-3 Expression by HA-LIP Attenuates Retinal Light Damage

**Figure 6:**
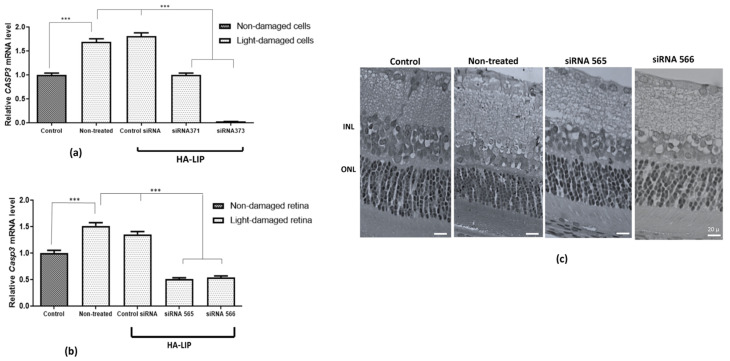
(**a**) In vitro CASP3 mRNA quantification in control non-treated damaged retinal cells and under siRNA 371 or siRNA 373 treatments by RT-qPCR. (**b**) In vivo Casp3 mRNA quantification in control non-treated damaged retinas and under siRNA 565 or siRNA 566 treatment by RT-qPCR. Results were expressed as mean ± standard error from values of RT-qPCR experiments and analyzed by 2-way analysis of variance (ANOVA) and Tukey’s post-test, which *p* < 0.05 (***) was considered significant. (**c**) Representative image of untreated damaged retina of albino Wistar rates by a single exposure to white LED light at 40,000 lux for 8 h, under the siRNA 565 or siRNA 566 treatments, compared with the control.

To verify the potential retinal protective effects of HA-LIP, each sequence of Stealth siRNA targeting caspase-3 was incorporated into lipoplexes and tested in the retinal light degeneration model. First, we tested the in vitro *CASP3* mRNA knockdown in ARPE-19 cells, after 8 h of LED light exposure at 2500 lux. According to the results, the untreated cells showed a significant increase in *CASP3* expression compared with the control (*p* < 0.05). Moreover, the HA-LIP complexed with the siRNA 373 showed the highest silencing level of the target gene compared with siRNA 371 (Figure 6a), a significant reduction of more than 97% of *CASP3* expression, compared with the untreated cells exposed to the LED lights (*p* < 0.05).

During the in vivo evaluation, the expressions of *Casp3* in retinal tissue increased after 8 h of retinal light exposure (*p* < 0.0001), compared with the control. The intravitreal injections of HA-LIP containing siRNA 565 or siRNA 566 significantly reduced *Casp3* expression (*p* < 0.0001) to 66.40% and 64.26%, compared with the untreated animals exposed to retinal light injury (Figure 6b). No significant variation in *Casp3* inhibition was observed between HA-LIP containing siRNA 565 and siRNA 566.

## 4. Discussion

This work aimed to obtain an efficient non-viral vector for the intravitreal delivery of siRNA, with easy preparation and high silencing efficiency. Previously, our research group developed a cationic system containing sodium alginate to increase the stability and effectiveness of lipid-based siRNA vectors [8]. However, for an intravitreal application, this positively charged system may not reach the retinal tissues due to the extended presence of negative charges in the vitreous humor [24,27,34]. Therefore, we tested the HA as an alternative coating strategy to facilitate the intraocular mobility of lipoplexes and siRNA internalization into retinal cells. In this experiment, we successfully obtained monodisperse nanoparticles with an anionic surface charge and a size in the range of the average diameter liposome formulations indicated in biomedical applications [5]. Regarding the lipoplexes’ morphology, TEM and cryo-TEM images showed spherical vesicles and size in accordance with DLS studies. As the overall charge and shape of lipoplexes are related to the structure of the lipid and conditional adjustments, an increase in lipoplex size and Zeta potential values in comparison with uncoated ones was associated with the electrostatic binding of HA onto the lipoplex surface. The irregular core of the cationic lipoplex (Figure 1a) is probably due to sample preparation before the TEM analysis.

Next, we investigated if the HA coating could interfere with siRNA encapsulation in lipoplexes (Figure 1b). In this study, electrophoresis was used to compare the band size of naked siRNA and the band size of siRNA released from nanoparticles after treatment with NaCl 1M and Triton-100X, which promoted lipoplex dissociation by solubilization of the lipids. Without the treatment addition, the siRNA band was not observed, indicating that siRNA molecules were indeed associated with the cationic lipoplex and HA-LIP (Figure 1b: band 03 and band 05). Moreover, the band of released siRNA from both nanoparticles after treatment (Figure 1b: band 04 and band 06) showed an electrophoretic migration comparable with and similar to the naked siRNA’s band migration (Figure 1b: band 01 and band 02). In the electrophoresis assay, we were not able to recover the total amount of siRNA used to prepare the lipoplexes after treatment with Triton X-100. Based on this, we suggest that Triton X-100 treatment in the electrophoresis assay decreased staining sensitivity of siRNA when the mixture was loaded onto gel or did not completely dissociate siRNA from the lipoplexes, as Hamoudi et al. previously described [6]. To determine the amount of siRNA encapsulated inside the liposome preparations, we used the RiboGreen Assay Kit. Our results showed that only a small amount of the siRNA molecules was not complexed with the lipoplex formulations, and the encapsulation efficiency of siRNA in the cationic lipoplex was 97.3%, and 96.6% in HA-LIP. Therefore, we suggest that the HA coating did not impair the incorporation and release of siRNA from the nanoparticles.

As shown in Figure 2d, HA-LIP could facilitate the intracellular delivery of siRNA into ARPE-19 cells, despite their negative superficial potential. The three-dimensional reconstruction of the confocal immunofluorescence images (Figure 2c,d) revealed a qualitative representation of siRNA internalization in ARPE-19 cells and the rhodamine-siRNA uptake experiment did not complete the 48 h of transfection as in the RT-qPCR in vitro assay. The uptake of HA lipoplex is believed to happen through the interaction between HA and the CD44 receptors of ARPE-19 cells, which facilitates the internalization in retinal cells [29,35]. Furthermore, the cellular viability of ARPE-19 cells incubated with cationic lipoplexes and HA-LIP was performed (Figure 2e). The treatment using HA-LIP induced a lower decrease in the metabolic activity of ARPE-19 cells, compared with cationic lipoplex, especially at 40 nM, which was statistically significant. This difference in cell viability may be due to one of HA’s numerous biological functions, which include the promotion of cell proliferation, migration and intracellular signaling [30].

RNAi therapy’s success relies on the siRNA delivery vector’s suitability to overcome intraocular barriers and reach the retinal tissues. Until now, questions have been raised about the feasibility of a cationic lipoplex electrostatically coated with HA to deliver siRNA in vivo. While some previous ex vivo studies suggested that the functionalization of lipoplex with HA is a promising alternative for this goal [24,25], another recent ex vivo study indicated that this strategy was not sufficient to ensure retinal penetration [36]. As both studies did not evaluate the HA-coated lipoplexes in vivo, in the present work, the cationic lipoplex was compared with the same system functionalized with HA in terms of siRNA delivery efficiency and safety after intravitreal injection. The retinal distribution assay results demonstrated that both lipoplexes could transport the siRNA through the intravitreal cavity and facilitate this nucleic acid’s internalization in retinal tissues a few hours after intravitreal injections. Despite the cationic charges, uncoated lipoplex could overcome the vitreous barrier and reach the ganglion cell layer. However, they could not penetrate deeper retinal layers, as shown in Figure 3a,b. On the other hand, the fluorescent siRNA delivered by HA-LIP could be seen in both the outer and inner retinal layers, and accumulated in the RPE. Interestingly, an important mechanism of the internalization of anionic nanoparticles into the deeper retinal structures can happen through the interaction with Müller cells [34]. Therefore, HA-LIP may be able to across the retina and reach the RPE through interaction with these retinal glial cells, but further studies must be carried out in this regard.

Not only the surface modification of HA-LIP but also the route of administration was essential for reaching this result. Previous studies showed that the bioavailability of nucleic acids in the posterior segment of the eye after topical instillation is poor, especially because of their size [37,38]. Systemic administration showed similar results and only 1–2% of the therapeutic molecules administered could reach the vitreous cavity, due to the existence of the blood–retina barrier [5,18]. In addition, retinal delivery systems could be accomplished using other routes such as the periocular (sub-conjunctival, sub-Tenon’s, peribulbar and retrobulbar injections) and suprachoroidal routes. However, these approaches may not result in adequate drug concentrations in the retina with the currently available technologies [14]. As intravitreal injections can be used for direct insertion of nucleic acids in the posterior segment of the eye, this administration provided good distribution and extensive uptake of this siRNA by retinal tissues. In order to investigate the safety of lipoplex after intravitreal injections in the retina, visual electrophysiological functional exams were combined with clinical evaluations and histologic assays. To assess the visual pathway of the retina, ERG exams were performed 7 and 14 days after the intravitreal injection of both lipoplexes. Considering the a -and b-waves’ amplitudes in both scotopic and photopic conditions of ERG, statistical analyses showed no significant changes in their values during the experiment (Figure 4). Contributing to these findings, the Naka–Rushton hyperbolic function results showed remarkably similar b-wave amplitude responses between treatment and contralateral eyes, when they were exposed to a progressive increase in light stimulus (Figure 4f). Based on our findings, intravitreal administration of cationic and HA-LIP may not affect rods’ and cones’ functionality.

These results were in accordance with the clinical evaluation of the rats’ retina, which showed no apparent toxicities of the lipoplex in vivo. To evaluate the internal ocular health, a fundus examination was carried out, and no damage changes were observed before and after intravitreal injections (Figure 5d). Regarding the IOP measurements, the rat group that received the HA-LIP and saline in the contralateral eye exhibited an expressive IOP reduction in both eyes at 14 days after the intravitreal injections, compared with the other group (Figure 5c). According to another work that established the normal range of IOPs in Lewis rats using the Tono-Pen tonometer, with a 90% confidence interval, the values were 7.28 mm Hg for the lower limit and 26.98 mm Hg for the higher limit [39]. Based on this, despite the IOP variations found in this animal group, these eyes had their main values in the normal range of rat IOPs (16.5 ± 5.4 for saline and 14.5 ± 3.4 for HA-LIP). Moreover, these authors reported that false IOP readings could occur after touching the eyelid or lacrimal meniscus, and because of the anesthesia. Since the contralateral eyes showed similar results at the same time of the experiment, the lower intraocular pressure may not be related to the toxic effect of lipoplex. Furthermore, the histological results did not show significant changes between the eyes that received lipoplex formulations and saline (Figure 5e). Based on these results, cationic lipoplex and HA-LIP were considered safe for intravitreal administrations. These findings agree with other histological studies of liposomes after intravitreal injection [40,41].

The investigation of HA-LIP gene silencing activity was first performed in ARPE-19 cells. Light-induced retinal damage was used as an experimental model to study retinal degeneration, particularly because it can be detrimental to retinal biochemistry and morphology. Generally, this retinal injury is characterized by degeneration and loss of the photoreceptor’s outer segments by apoptosis or necrosis [42,43,44]. However, the complexity underlying the molecular mechanisms are still unclear and require further investigations. We selected *CASP3* because it is a cysteine protease recognized as one of the key signaling proteins of apoptosis, involved in both intrinsic and extrinsic apoptotic pathways [45,46]. The data reported here showed that *CASP3* expression was significantly induced upon light insult, compared with the control. Furthermore, an efficient gene silencing was shown in both treatments with HA-LIP containing Stealth sequences, particularly the nanoparticles containing siRNA 373 (Figure 6a). These results highlight the good properties of HA lipoplex as a non-viral vector of siRNA and stimulate further studies to evaluate in vivo applications, as it is important to test the new delivery systems in retinal models that better mimic the outer blood–retina barrier [47].

To evaluate if HA-LIP containing specific siRNAs targeting *Casp3* could protect theretina against the light degeneration model, we applied these formulations through intravitreal injection 1 day before the light injury. Since apoptosis is the main mechanism of retinal cell death in induced and inherited retinal degenerations, such as ganglion cells in glaucoma and photoreceptors in age-related macular degeneration (AMD), it is expected that *Casp3* is involved in this pathogenesis [48]. Indeed, in retinal light damage in rats, the activation of different caspases (1, 3, 7, 8, and 9) has been reported, and it depends on the animal model and light levels used [42,45]. After retinal degeneration, untreated groups showed morphological changes in retinal tissues with a decrease in ONL thickness when compared with the control group. In contrast, the ONL thickness was preserved in treated groups (Figure 6c). The improved retinal morphology found after in vivo HA-LIP treatments (siRNA 565 and 566) suggested that Casp3 gene silencing promoted by the delivered siRNA (Figure 6b) could protect the photoreceptors and attenuate the injury-induced after excessive LED light exposure. In fact, the advanced gene knockdown observed in vivo may have resulted from enhanced penetration and displacement of HA-LIP in the retinal structure, as can be seen in Figure 3. This increased internalization of siRNA into retinal tissues confirmed that the functionalization of the lipoplex with HA led to a significant delivery of siRNA to retinal layers, with to the cationic lipoplex. As *Casp3* plays an important role in retinal injury induced by LED lights, *Casp3* siRNA contained in HA-LIP represents a safe and potential nanocarrier formulation for the treatment of retinal degeneration.

## 5. Conclusions

A novel lipid-based delivery vector was developed to protect siRNA on the journey to the retinal tissue and to promote gene silencing towards therapeutic RNAi effects. In this work, we demonstrated, for the first time, in vivo gene silencing in the retina using HA-LIP to deliver siRNA to retinal cells. Consequently, the inhibition of *Casp3* expression attenuated retinal degeneration after excessive LED light exposure. Notably, the intravitreal injections of lipoplex did not induce damage or induce toxic effects that could impair the function and morphology of the rat retina. Overall, our results indicate that HA-LIP can successfully deliver siRNAs to reduce the production of proteins involved in the apoptotic cascade of retinal neurodegeneration. Here, we open new possibilities for the treatment of many retinal diseases using non-viral vectors carrying siRNA, especially disorders that involve photoreceptor death.

## Figures and Tables

**Table 1:** siRNA sequences targeting caspase-3. Sense strands are presented.

Stealth	siRNA Sense Sequences Targeting Caspase-3
siRNA 371	5′-GGA AUA UCC CUG GAC AAC AGU UAU A-3′
siRNA 373	5′-GGU GGC AAC AGA AUU UGA GUC CUU U-3′
siRNA 565	5′-GGA CAA CAA CGA AAC CUC CGU GGA U-3′
siRNA 566	5′-CCU UAC UCG UGA AGA AAU UAU GGA A-3′

**Table 2:** Sequences of forward and reverse primers used in the RT-qPCR assays.

Gene and Accession Number (GenBank)	Sequence (5′-3′)	Amplicon Size (pb)
*ACTB_Human* (NM_001101.5)	F:AGAGCTACGAGCTGCCTGAC R:AGCACTGTGTTGGCGTACAG	184
*CASP3_Human* (NM_004346.4)	F:TCTTGGCGAAATTCAAAGGATGG R:GTAGCGTCAAAGGAAAAGGACTC	152
*Actb_Rattus* (NM_031144.3)	F: AGACCTCTATGCCAACACAGTG R: TAGAGCCACCAATCCACACAG	161
*Casp3_Rattus* (NM_012922.2)	F: GGAAGATCACAGCAAAAGGAGC R: CAGTAGTCGCCTCTGAAGAAAC	129

*ACTB* (*Actb*): actin, beta; *CASP3* (*Casp3*): caspase 3; bp: base pairs. RT-qPCR assays were performed to evaluate the mRNA levels of the caspase-3 gene. *ACTB* (actin, beta) gene was chosen as a reference gene for the normalization of the data [32].

**Table 3:** Size, PDI and zeta potential of lipoplexes in 150 mM NaCl, as measured by dynamic light scattering.

Lipoplex Formulation	Z-Averaged Diameter (nm)	PdI *	Zeta Potential (mV)	EE (%)
Cationic lipoplex	133.5 ± 1.2	0.217 ± 0.014	71.1 ± 3.0	97.3%
HA-LIP (160–600 kDa)	221.8 ± 9.2	0.220 ± 0.068	−34.2 ± 1.4	96.6%

* PdI: Polydispersive index.

## Data Availability

The data presented in this study are available on request from the corresponding author.
